# In-depth biological analysis of alteration in *Plasmodium knowlesi*-infected red blood cells using a noninvasive optical imaging technique

**DOI:** 10.1186/s13071-022-05182-1

**Published:** 2022-03-02

**Authors:** Moh Egy Rahman Firdaus, Fauzi Muh, Ji-Hoon Park, Seong-Kyun Lee, Sung-Hun Na, Won-Sun Park, Kwon-Soo Ha, Jin-Hee Han, Eun-Taek Han

**Affiliations:** 1 Department of Medical Environmental Biology and Tropical Medicine, Kangwon National University School of Medicine, Chuncheon, Gangwon-do 24341 Republic of Korea; 2 Department of Obstetrics and Gynecology, Kangwon National University School of Medicine, Chuncheon, Gangwon-do 24341 Republic of Korea; 3 Department of Physiology, School of Medicine, Kangwon National University, Chuncheon, Gangwon-do 24341 Republic of Korea; 4 Department of Molecular and Cellular Biochemistry, Kangwon National University School of Medicine, Chuncheon, Gangwon-do 24341 Republic of Korea

**Keywords:** Imaging technique, Holotomography, *Plasmodium knowlesi*, Hemoglobin, Diffraction optical tomography (DOT), 3D refractive index, Membrane fluctuation, Host cell

## Abstract

**Background:**

Imaging techniques are commonly used to understand disease mechanisms and their biological features in the microenvironment of the cell. Many studies have added to our understanding of the biology of the malaria parasite *Plasmodium knowlesi* from functional in vitro and imaging analysis using serial block-face scanning electron microscopy (SEM). However, sample fixation and metal coating during SEM analysis can alter the parasite membrane.

**Methods:**

In this study, we used noninvasive diffraction optical tomography (DOT), also known as holotomography, to explore the morphological, biochemical, and mechanical alterations of each stage of *P. knowlesi*-infected red blood cells (RBCs). Each stage of the parasite was synchronized using Nycodenz and magnetic-activated cell sorting (MACS) for *P. knowlesi* and *P. falciparum*, respectively. Holotomography was applied to measure individual three-dimensional refractive index tomograms without metal coating, fixation, or additional dye agent.

**Results:**

Distinct profiles were found on the surface area and hemoglobin content of the two parasites. The surface area of *P. knowlesi*-infected RBCs showed significant expansion, while *P. falciparum*-infected RBCs did not show any changes compared to uninfected RBCs. In terms of hemoglobin consumption, *P. falciparum* tended to consume hemoglobin more than *P. knowlesi*. The observed profile of *P. knowlesi*-infected RBCs generally showed similar results to other studies, proving that this technique is unbiased.

**Conclusions:**

The observed profile of the surface area and hemoglobin content of malaria infected-RBCs can potentially be used as a diagnostic parameter to distinguish *P. knowlesi* and *P. falciparum* infection. In addition, we showed that holotomography could be used to study each *Plasmodium* species in greater depth, supporting strategies for the development of diagnostic and treatment strategies for malaria.

**Graphical Abstract:**

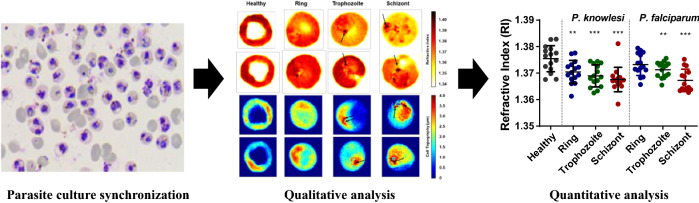

**Supplementary Information:**

The online version contains supplementary material available at 10.1186/s13071-022-05182-1.

## Background

Malaria pathophysiology remains an interesting topic for exploration. Although many studies have been conducted, the wide variety of *Plasmodium* species with features such as immune evasion and drug resistance make malaria difficult to eradicate [1–3]. Imaging techniques have long been used in malaria pathophysiology studies. Since the introduction of the first light microscope, it has continuously evolved into exceptionally sophisticated technology such as Förster resonance energy transfer (FRET), fluorescence lifetime imaging microscopy (FLIM), third-harmonic generation (THG), two-photon absorption fluorescence (2PAF) imaging, and common-path diffraction optical tomography (cDOT) [4].

A noninvasive optical technique, digital holotomography, has been used to explore the morphological, biochemical, and mechanical changes during the development of *Plasmodium falciparum* and *Babesia* parasite-infected red blood cells (RBCs) [5, 6]. Holotomography uses optical interferometry or digital holography, which is noninvasive, label-free, and quantifiable. In addition, cDOT is a recent technique that measures both the three-dimensional (3D) structure and dynamics of live cells simultaneously [6, 7].

Out of six *Plasmodium* species that inflect humans, *P. knowlesi* is considered to be a pathogen of greatest threat. It is recognized as zoonotic malaria particularly in Malaysia and has spread to other areas in Southeast Asia [8, 9]. During the host cell invasion, the parasite modifies the host cell environment to support its life, leading to morphological, biochemical, and mechanical modification [10, 11].

A recent study of *P. knowlesi* topography applied serial block-face scanning electron microscopy (SEM) for structural observation [12]. However, the technique required a fixation and metal cover before analysis, which is a drawback in determining the live dynamics of parasites and host cells [6, 13, 14]. Instead of using an additional treatment, our study focuses on live-cell biological analysis. Thus, the cells are kept in their native form without undermining the cell membrane during observation. In addition, interferometric microscopy, holotomography, is considered an easy and fast technique that reduces time and cost by skipping the labeling, metal cover, and cell fixation steps [15, 16].

Holotomography can measure the refractive index (RI) of tremendously different cells. RI is an intrinsic optical profile that can be used as a key parameter for measuring the biophysical alteration of an object [17]. Since RI can distinguish abnormal cells, it has been applied for diagnostics in several disciplines including cell biology, hematology, and pathology [18]. It also was reported that it could be used as an intrinsic marker for cancer diagnosis [19]. Differences in values from abnormal cells are used to standardize the level of severity by integrating it into the machine learning model [20]. Thus this technique may become a key clinical decision support tool.

Here we present label-free 3D imaging and quantification of *P. knowlesi*-infected RBCs compared to *P. falciparum*-infected RBCs. We explore both parasites' characteristics with regard to the RI, cytosol volume, hemoglobin content, sphericity, surface area, and fluctuation. Similar patterns are observed between our study and others that use different approaches, proving that this technique is unbiased [5, 6, 12]. A striking difference between the two parasites was observed in surface area and hemoglobin. Expansion of the surface area of *P. knowlesi* was observed in the infected RBCs, while *P. falciparum* tended to be unchanged compared to uninfected RBCs, as reported previously [12]. In terms of hemoglobin content, *P. knowlesi* consumes less hemoglobin than *P. falciparum*. Thus the observed profile of the surface area and hemoglobin content of parasite infected-RBCs can be used as a diagnostic parameter to distinguish *P. knowlesi* and *P. falciparum* infection. In addition, our study demonstrates that this technique could also be used for analyzing a broad range of *Plasmodium* species, thus potentially contributing to the development of diagnostic and treatment strategies as well as for pathophysiological study.

## Methods

### Parasite culture

*Plasmodium knowlesi* A1.H1-adapted human RBCs were cultured in RPMI 1640 medium (Invitrogen Life Technologies, Grand Island, NY) supplemented with horse serum (Gibco, Life Technologies), L-glutamine, 25 mM HEPES (Invitrogen Life Technologies), and 0.5% AlbuMAX II (Invitrogen Life Technologies). The culture was maintained at 2% hematocrit with mixed gas (90% N_2_, 5% CO_2_, and 5% O_2_) at 37 °C, while *P. falciparum* was cultured without serum and supplemented with AlbuMAX I instead of AlbuMAX II [21]. Both parasites were treated with gentamicin to prevent contamination.

*Plasmodium knowlesi* schizonts were isolated using 50% Nycodenz, while *P. falciparum* 3D7 strain parasites were used a magnetic-activated cell sorting (MACS) system [22]. Purified schizonts were re-cultured into supplemented media as described above with initial 2% parasitemia. The ring stage of *P. knowlesi* was observed 10 h after re-culture, and that of *P. falciparum* approximately 18 h [23, 24]. The parasite development was monitored and then sorted according to stage for further examination.

### Analysis of alteration in parasite-infected human RBCs

Three to five percent parasitemia of each parasite stage (ring, trophozoite, and schizont) was set for analysis using a commercial 3D cDOT system (HT-1H, Tomocube, Inc., Republic of Korea). The parasite was diluted with Dulbecco’s phosphate-buffered saline (DPBS) without calcium and magnesium, then placed in a 25 × 50 mm coverslip (Matsunami Glass Ind., Ltd., Osaka, Japan). The data were visualized using TomoStudio software (Tomocube, Inc.) and analyzed quantitatively by RBC characterization software using a specific algorithm [16]. A total of 15 infected RBCs from each stage were used for analysis.

### Statistical analysis

GraphPad Prism (GraphPad Software, San Diego, CA, USA) was used for statistical data analysis. The Mann–Whitney test was applied to determine differences between the two groups. A value of *p* < 0.05 was considered to indicate a significant difference. All of the data values in the text with ± indicate standard deviations.

## Results

### Three-dimensional refractive index maps of malaria-infected RBCs

**Fig. 1:**
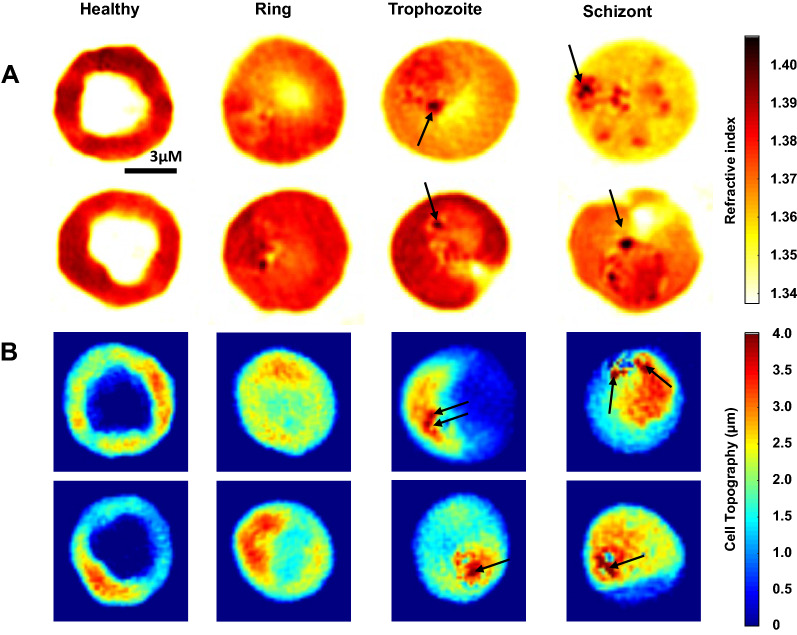
Three-dimensional refractive index and phase map of *P. knowlesi*-infected RBCs. **a** 3D RI maps of *P. knowlesi*-infected RBCs and **b** cell topography shown by 2D phase map image. The black arrow indicates hemozoin. Images in rows from the left to right show uninfected RBCs, ring, trophozoite, and schizont using focused plane (top). Color maps show the refractive index (*n*) (top right) and the cell topography (bottom right). The scale bar is 3 μm. u.RBCs = uninfected RBCs. The 3D-rendered isosurface of RI maps from the top view

**Fig. 2:**
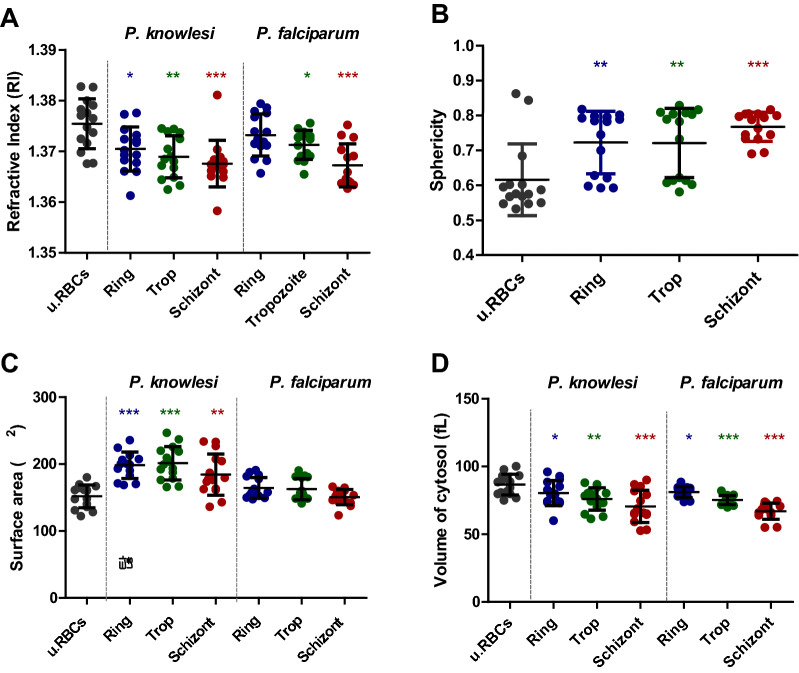
Refractive index and morphological profile of *P. knowlesi*-infected RBCs. **a** Refractive index, **b** sphericity, **c** cellular volume, and **d** surface area of *P. knowlesi-*infected RBCs. Each data point represents 15 samples of uninfected RBCs and *P. knowlesi*- and *P. falciparum*-infected RBCs. The asterisks indicate the statistical significance (*P* < 0.05) of the ring, trophozoite, and schizont compared to uninfected RBCs. The bar indicates a mean value and standard deviation. u.RBCs = uninfected RBCs

The RI is commonly used for analyzing various features of cells. it serves as an intrinsic optical imaging contrast for 3D optical imaging [17]. Each specific blood stage of the malaria parasite was observed after synchronization (Additional file 1: Figure S1). The RI of *P. knowlesi*-infected RBCs of the ring (1.370 ± 0.004), trophozoite (1.369 ± 0.004), and schizont (1.368 ± 0.004) decreased significantly as compared to the uninfected RBCs (1.375 ± 0.005). The profile differences are described in the RI and phase map images (cell topography) of each developmental stage compared to uninfected RBCs (Fig. 1). A similar pattern was seen in *P. falciparum* RI, with a slightly less significant reduction than *P. knowlesi* in the ring (1.373 ± 0.004), trophozoite (1.371 ± 0.003), and schizont (1.367 ± 0.004) stages (Fig. 2a).

### Morphological alteration of *P. knowlesi*-infected RBCs in different stages

Parasite growth during infection induces host cell deficiency [25], which was seen in *P. knowlesi*-infected RBCs. A morphological change was observed from the initial biconcave or donut-like shape to more spherical. The sphericity of *P. knowlesi*-infected ring (0.723 ± 0.087), trophozoite (0.721 ± 0.096), and schizont (0.768 ± 0.040) was significantly greater compared to uninfected RBCs (0.616 ± 0.099) (Fig. 2b). The surface area of the RBCs was significantly expanded compared to uninfected RBCs (151.821 ± 16.558 μm^2^) following the development stage of the ring (198.408 ± 19.020 μm^2^), trophozoite (201.281 ± 23.982 μm^2^), and schizont (184.317 ± 29.821 μm^2^) stages (Fig. 2c). However, the schizont surface area of *P. knowlesi*-infected RBCs showed a slight decrease (Fig. 2c). In terms of *P. falciparum*-infected RBC surface area, there was no change compared to uninfected RBCs (mean value for the ring of 164.320 ± 14.919 μm^2^, trophozoite of 162.540 ± 15.273 μm^2^, and schizont of (150.702 ± 11.053 μm^2^ (Fig. 2c).

A reduction in cytosol volume was observed during parasite infection. The parasite occupied and consumed a provided nutrient in the cytosol. The mean value for the ring (80.413 ± 9.015 fl), trophozoite (76.084 ± 8.001 fl), and schizont (70.558 ± 11.484 fl) showed a significant decrease compared to uninfected RBCs (86.686 ± 7.360 fl) (Fig. 2d). A similar pattern of cytosol volume was found for *P. falciparum*, which exhibited a significant reduction to 81.182 ± 3.828, 75.323 ± 3.115, and 66.999 ± 15.730 fl for the ring, trophozoite, and schizont, respectively. The trophozoite stage of *P. falciparum* was observed to have more impact on the cytosol volume of RBCs than the trophozoite of *P. knowlesi*.

### Membrane fluctuation in asexual stages of *P. knowlesi*-infected RBCs

**Fig. 3:**
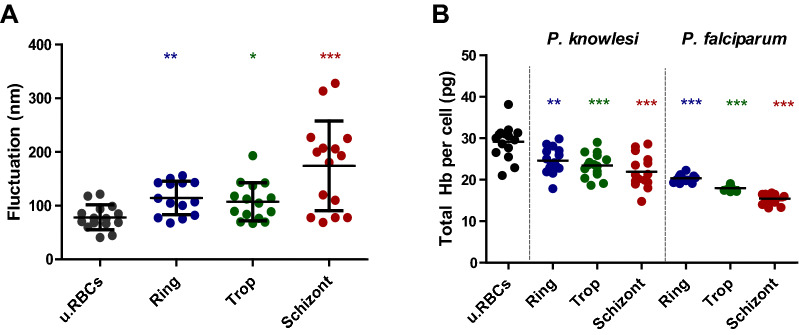
Membrane dynamic and biochemical alteration of *P. knowlesi* and *P. falciparum*-infected RBCs. **a** Fluctuation of each stage in *P. knowlesi*-infected RBCs and hemoglobin content of both *P. knowlesi*- and *P. falciparum*-infected RBCs. Each data point represents 15 samples of uninfected RBCs and *P. knowlesi*- and *P. falciparum*-infected RBCs. The asterisks indicate the statistical significance (*P* < 0.05) of the ring, trophozoite, and schizont compared to uninfected RBCs. The bar indicates a mean value and standard deviation. u.RBCs = uninfected RBCs

Since the parasite actively proliferates and grows within the RBCs, the structure of the host RBC membrane is affected by that process [26]. The infected RBCs, ring (114.634 ± 29.929 nm), and schizont (174.186 ± 80.668 nm) showed a significant increase in fluctuation rate compared to uninfected RBCs (78.330 ± 22.270 nm). However, the value for the trophozoite (107.395 ± 34.241 nm) seemed to decrease slightly from the ring and schizont stages (Fig. 3a).

### The hemoglobin content of *P. knowlesi*-infected RBCs

Hemoglobin is a major constituent of cytoplasm, and is used by the parasite as the nutrient for their growth and proliferation [27, 28]. The mean hemoglobin content of the ring (24.623 ± 3.047 pg), trophozoite (23.446 ± 2.814 pg), and schizont (21.950 ± 3.804 pg) was significantly lower than that in uninfected RBCs (29.143 ± 3.964 pg) (Fig. 3b). Moreover, *P. falciparum*-infected RBCs showed a more significant decrease compared to uninfected and *P. knowlesi*-infected RBCs following the development of the parasite (20.353 ± 0.822 pg, 17.968 ± 0.528 pg, 15.438 ± 1.171 pg, for the ring, trophozoite, and schizont, respectively).

## Discussion

Once malaria parasites hijack the RBCs, their morphology is actively altered following their growth and development [29]. Malaria parasites transform during the asexual stage from immature rings and trophozoites to the mature schizont stage. The RI is currently widely used for biological samples, including RBCs, to determine disease correlations. It is applicable not only for cell biology but also for hematology and pathology studies [18].

Every cell has relative variations in organelles, size, and protein content. This results in differences in the RI [30], which also applies to any biological alteration. In terms of malaria-infected RBCs, the RI drastically decreases, since the parasite growth filling the host RBCs induces cellular perturbation [31].

The dark red color indicates the highest RI value, while yellow indicates low RI. The uniformity of uninfected RBC content was described as uniform color, as most of the RBCs contain 95% spherical protein like hemoglobin [32], whereas the parasitized RBCs revealed a gradient color because of RBCs occupied by a parasite. As the parasite matures, a greater number of merozoites exist within the cells, and the greater the RI reduction within the cells. This is caused by several factors including the parasite vacuole occupying the cytoplasm, hemoglobin consumption, hemozoin formation, and export of parasite protein content [5].

The environment within the host RBCs generally consists of protein cytoplasm or hemoglobin [33], which continues to decline as the parasites grow, to support their metabolism. The parasites use hemoglobin as a precursor of amino acid synthesis [34]. Hemoglobin is degraded to the toxic product called hematin and is then converted to a structural compound called hemozoin to avoid reactive oxygen species [35, 36]. Hemozoin can be seen by the dark red color in the RI map. Hemozoin was also reported to produce high-contrast imaging observed using polarization microscopy, dark-field microscopy, and resonance Raman microscopy [4, 37, 38].

Different phenotypic profiles of *P. knowlesi* and *P. falciparum* generated differences in RI and host morphology. The size of *P. knowlesi* merozoites is larger (~ 2–3 um) than that of *P. falciparum* (~ 1–1.5 um), which might contribute significantly to RI differences [39] Thus, *P. falciparum* RI exhibited a slightly less significant reduction compared to *P. knowlesi*.

The surface area of infected RBCs and cytosol volume exhibited an inverted ratio to the RI, since *P. falciparum* produces up to 30 merozoites, which is greater than *P. knowlesi*, which only reaches a maximum of 16 merozoites [40]. The number of each merozoite might contribute more to a characteristic of the surface area and cytosolic volume of the infected RBCs. The increased surface area of infected RBCs is caused by the addition of the membrane-like parasite vacuole membrane (PVM) that is used by the parasite for nutrient uptake [41]. However, the schizont-infected RBC surface area was slightly decreased from other parasite stages, which may be due to the schizont arrangement to burst out from the host cells with optimization of their ratio of the surface to volume. In terms of *P. falciparum*-infected RBCs, the surface tended to remain unchanged at all stages of the parasite compared to uninfected RBCs. This leads to the possibility that the *P. falciparum* fusion vesicle membrane has insufficient phospholipids to drive surface area expansion [12] because of the reorganization of compact spectrin oligomers [42].

Increasing the sphericity of the infected RBCs leads to the loss of the donut shape. This is because the parasite induces the modification of RBCs by expression of a particular parasite protein into the RBC membrane, resulting in alteration of the RBC geometry [43]. The loss of the donut-like shape causes a reduction in the RBC surface area-to-volume ratio, making their shape more spherical and leading to the loss of deformability [43, 44]. The donut shape has a highly flexible membrane, and the high surface area to volume facilitates a huge reversible elastic deformability to pass through the narrowest blood vessels [43]. Once RBC deformability is disrupted, the malaria parasite can induce infected RBC adherence to the vascular endothelium of several organs, called sequestration, as an immune evasion mechanism [45]. This leads to severe malaria and then to death caused by organ failure through blood vessel blockage [46].

The membrane fluctuation of infected RBCs was significantly elevated following parasite behavior. The highest fluctuation occurred during the schizont stage when the parasite prepared merozoite egress. Moreover, the malaria parasite released various proteins that help merozoites burst out from the RBC membranes. A major protein released is a protease such as cytoskeleton-degrading malarial proteases, falcipain-2, and plasmepsin II, and also a family of putative papain-like proteases called serine repeat antigen [47, 48]. This results in a high fluctuation in the RBC membrane [5]. Several excess nutrients are also transferred by several pathways including new permeability pathways that are well known and studied in *P. falciparum* [49]. Meanwhile, the fluctuation of the ring stage is higher than the trophozoite from the arrangement of a new host RBC environment such as food vacuole formation originated from Maurer’s clefts [50]. This process required numerous proteins such as membrane-associated histidine-rich protein 1 (MHRP-1), the skeleton-binding protein 1 (SBP1), ring-exported protein-2, knob-associated histidine-rich protein (KAHRP), erythrocyte membrane protein-like PfEMP, and ring-exported protein 1 (REX1) [51].

Our study focused on the RBCs, while previous research has focused more on the parasite alteration itself [12]. This study is also comparable to previous research that used a similar technique in *P. falciparum* and *Babesia* parasites. Our study found that the unique nature of *P. knowlesi* did not cause a significant change in hemoglobin content as compared to *P. falciparum*. Possible reasons for the higher hemoglobin content of *P. knowlesi*-infected RBCs than *P. falciparum* are as follows: (1) The *P. knowlesi* life-cycle is shorter than *P. falciparum*; thus, *P. knowlesi* did not have enough time to consume the hemoglobin. (2) Host preference differences: regulation of hemoglobin metabolism in *P. knowlesi* which naturally infects macaques RBCs may differ in the way they infect human RBCs. (3) The parasite might use another source more than hemoglobin. (4) *Plasmodium falciparum* has more merozoites than *P. knowlesi*.

In addition, clinical data have strengthened this assumption, where *P. knowlesi* hemoglobin levels were higher in patients than *P. falciparum*, with 9.7 g/dl reported for *P. knowlesi* and 7.15 g/dl for *P. falciparum* [52]. This raises the possibility that *P. knowlesi* treatment against hemoglobin metabolism may not have much more affect than *P. falciparum.* However, this hypothesis requires further study, particularly using clinical samples.

## Conclusion

In conclusion, the observed morphological, biochemical, and mechanical modification contributes to the pathophysiological study. Our study demonstrated that this noninvasive technique could be used for broad *Plasmodium* species analysis. In addition, the observed profile revealed that each species has different pathways to support their life-cycles. Thus, understanding the characteristics of each *Plasmodium* species contributes to developing diagnostic and treatment strategies against the malaria parasites.

## Supplementary Information

**Additional file 1: Figure S1.** Morphology of malaria parasite during synchronization. (**a**–**c**) *Plasmodium knowlesi* culture*,* (**d**–**f**) *P. falciparum* culture. (**a** and **b**) mixed stages of parasites, (**b** and **e**) synchronized schizont stage, and (**d** and **f**) newly invaded ring stage. *Plasmodium knowlesi* synchronized schizont requires 10 h to reach the ring stage, while *P. falciparum* needs approximately 18 h.

## Data Availability

The datasets supporting the conclusions are included in the article.

## Abbreviations

SEM: Scanning electron microscopy
RBCs: Red blood cells
FRET: Förster resonance energy transfer
FLIM: Fluorescence lifetime imaging microscopy
THG: Third-harmonic generation
2PAF: Two-photon absorption fluorescence
cDOT: Common-path diffraction optical tomography
*P. falciparum*: *Plasmodium falciparum*
*P. knowlesi*: *Plasmodium knowlesi*
MACS: Magnetic-activated cell sorting
DPBS: Dulbecco’s phosphate-buffered saline
RI: Refractive index
PVM: Parasite vacuole membrane
MHRP-1: Membrane-associated histidine-rich protein 1
SBP1: Skeleton-binding protein 1
KAHRP: Knob-associated histidine-rich protein
REX1: Ring-exported protein 1
