# Supplementary figures and images for: In-depth biological analysis of alteration in Plasmodium knowlesi-infected red blood cells using a noninvasive optical imaging technique
Source: Parasit Vectors. 2022 Mar 2;15:68. doi: 10.1186/s13071-022-05182-1 (PMC8889714; doi:10.1186/s13071-022-05182-1)

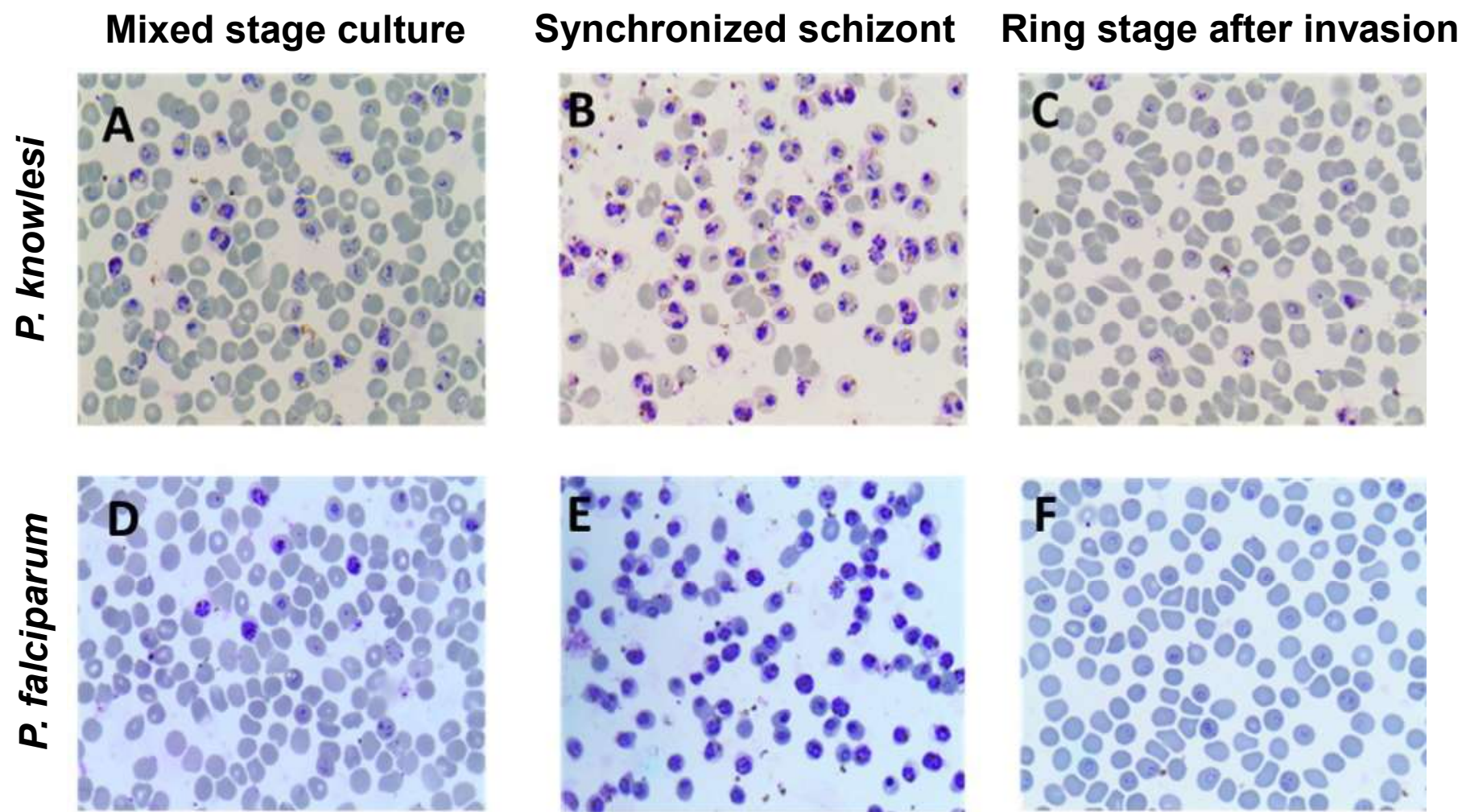

Additional file 1: Figure S1.

Supplement: Supplementary file 1 — Additional file 1: Figure S1. Morphology of malaria parasite during synchronization. (a–c) Plasmodium knowlesi culture, (d–f) P. falciparum culture. (a and b) mixed stages of parasites, (b and e) synchronized schizont stage, and (d and f) newly invaded ring stage. Plasmodium knowlesi synchronized schizont requires 10 h to reach the ring stage, while P. falciparum needs approximately 18 h. [file 13071_2022_5182_MOESM1_ESM.pdf]
